# Muscle metabolic stress determines cancer cachexia severity in mice

**DOI:** 10.3389/fphys.2022.1033932

**Published:** 2022-11-28

**Authors:** Christiano Alves, Laurie Goodyear, Patricia Brum

**Affiliations:** ^1^ School of Physical Education and Sport, University of Sao Paulo, Sao Paulo, Brazil; ^2^ Joslin Diabetes Center, Harvard Medical School, Boston, MA, United States

**Keywords:** muscle wasting, atrophy, oxidative metabolism, oxidative stress, lung carcinoma

## Abstract

**Objectives:** To determine the metabolic effects of cancer-conditioned media on myotube metabolism and to understand whether the variability of these effects is associated with cancer cachexia progression.

**Materials and methods:** We established single-cell clones from murine Lewis lung carcinoma (LLC) cells and generated conditioned media from each clonal line. Differentiated primary mouse myotubes were incubated with conditioned media derived from each individual clonal cell line. After initial analysis, we selected a specific LLC clonal cell line that failed to induce metabolic stress in myotubes for further investigation *in vitro* and *in vivo*.

**Results:** Short-term incubation with conditioned media from 10/34 LLC clonal cells failed to affect oxygen consumption rate (OCR) in myotubes. Incubation with parental LLC-conditioned media decreased protein content and changed the expression of key regulators of muscle function in myotubes, but the incubation of conditioned media from a selected clone that failed to affect OCR in myotubes also did not affect protein content and expression of muscle regulators. Mice injected with parental LLC cells had a significantly reduced body mass and muscle wasting compared to the mice injected with cells derived from this selected LLC clone.

**Conclusion:** Factors secreted by LLC cells induce metabolic stress in primary myotubes and induce cancer cachexia in mice. However, a selected clonal LLC cell line that failed to induce metabolic stress in myotubes also promoted weaker catabolism in mice. These novel findings establish that early disruption of muscle oxidative metabolism is associated with cancer cachexia progression.

## Introduction

Cancer cachexia is characterized by progressive skeletal muscle wasting and is associated with a poor prognosis in several cancer types (Fearon et al., 2011; Fearon et al., 2012; Fearon et al., 2013; Kimura et al., 2015). Hypermetabolism is commonly observed at the early stages of cancer cachexia progression and disruption of muscle oxidative metabolism may be a trigger of muscle catabolism (Fearon et al., 2012; Dev et al., 2015; Vazeille et al., 2017; Jouinot et al., 2020). Our group and others have previously demonstrated that improved muscle oxidative metabolism induces resistance to cancer-induced muscle atrophy (Springer et al., 2012; Alves et al., 2020; Alves et al., 2021), but the complex crosstalk between different tumors and skeletal muscle during the early onset of cachexia syndrome is still not fully understood. Additional efforts and novel approaches to understanding the implications of this tumor-muscle relationship are still necessary.

Here, we applied a combination of cell cloning and extracellular flux experiments to determine the metabolic effects of cancer-secreted factors directly in myotubes. Since the progression of cancer cachexia is usually a heterogeneous process, we have established multiple clonal cell lines from Lewis lung carcinoma (LLC) and B16-F10 melanoma (B16) cells, which are two models commonly used to study cancer cachexia in mice (Das et al., 2011; Ruas et al., 2012; Petruzzelli et al., 2014; Alves et al., 2019). Our main results are derived from experiments with the LLC cells since their conditioned media clearly challenged the myotubes’ metabolism. Short-term incubation (12 h) with LLC-conditioned media increased basal OCR in primary myotubes, an indicator of acute metabolic stress. Long-term incubation (48 h) with LLC-conditioned media decreased protein content and changed the expression of key regulators of muscle function in myotubes. Remarkably, a selected LLC clonal cell line that failed to affect OCR in myotubes also failed to promote changes in protein content and expression of muscle regulators. Injection of this specific LLC clonal cell line in mice did not induce body catabolism as observed by injecting the parental LLC cells. This study demonstrates that early disruption of muscle oxidative metabolism is associated with cancer cachexia progression.

## Materials and methods

This study was approved by the Ethical Committee of the School of Physical Education and Sport at the University of Sao Paulo and by the Joslin Diabetes Center Institutional Animal Care and Use Committee (IACUC). All procedures were performed in accordance with NIH guidelines. Primary mouse myoblasts were isolated and differentiated into myotubes as previously described (Megeney et al., 1996a; Megeney et al., 1996b). B16 and LLC cells (ATCC, United States) were cultured in DMEM supplemented with 10% fetal bovine serum and 1% pen/strep. Clonal cell lines were established by picking single cells after serial dilutions in 96-well plates. Experiments were conducted with the second cell passage after original single clones were selected. To collect conditioned media, B16 and LLC cells were plated, reached a 50% confluency, and then were incubated in DMEM serum-free media for 24 h. Each cell-conditioned media was mixed with fresh media (ratio 1:1) and supplemented with 5% horse serum and then added to primary mouse myoblast-derived myotubes for 12 h (for metabolic assays) or 48 h (for protein content). Myotubes were washed with saline before any readout or extraction. Human skeletal myoblasts (Thermo Fisher Scientific; A11440) were differentiated into myotubes in Dulbecco’s modified Eagle’s medium (DMEM; Gibco) supplemented with 2% horse serum and 1% pen/strep as previously described (Alves et al., 2020).

Oxygen consumption rate (OCR) was measured in myotubes using extracellular flux analysis (XF96, Agilent Seahorse, MA United States) in sodium bicarbonate-free DMEM supplemented with 31.7 mM NaCl, 10 mM glucose, and 2 mM glutamax (pH 7.4 adjusted using NaOH). Protein content was determined with Pierce™ BCA Protein Assay Kit (ThermoFisher, United States). Reduction of the MTS tetrazolium compound was determined with CellTiter 96 AQueous One Solution Cell Proliferation Assay (MTS) (Promega, United States). To determine the expression of key regulators of muscle function, RNA was isolated, reverse transcribed and amplified as previously described (Alves et al., 2020). For each gene, mRNA expression was calculated relative to *Hprt* expression, which showed similar Ct values across samples. Primer sequences are *Hprt* F: AGT​CCC​AGC​GTC​GTG​ATT​AG; *Hprt* R: TTT​CCA​AAT​CCT​CGG​CAT​AAT​GA; *Fbxo32* F: TCA​GAG​AGG​CAG​ATT​CGC​AA; *Fbxo32* R: GGG​TGA​CCC​CAT​ACT​GCT​CT; *MyoD* F: CGG​GAC​ATA​GAC​TTG​ACA​GGC; *MyoD* R: TCG​AAA​CAC​GGG​TCA​TCA​TAG​A; *Ppargc1a* F: TGA​TGT​GAA​TGA​CTT​GGA​TAC​AGA​CA; *Ppargc1a* R: GCT​CAT​TGT​TGT​ACT​GGT​TGG​ATA​TG.

For *in vivo* experiments, twelve‐week‐old male C57BL/6 mice were used. Mice were injected subcutaneously with 10^5^ LLC cells diluted in serum-free DMEM medium as previously described (Alves et al., 2019). Tumor size, body mass, and cumulative food intake were assessed, and mice were euthanized 21 days post tumor cell injection by cervical dislocation under isoflurane anesthesia. Tumors were harvested and weighted, and tumor mass was subtracted from the final carcass mass to determine delta changes in body mass. Gastrocnemius and plantaris muscle from the left leg (opposite to the right flank injected with tumor cells) were also harvested, weighted, and normalized by initial body mass (pre tumor injections) to determine skeletal muscle mass.

Data analyses were conducted using Graph Pad Prism 8 (Graph Pad Software Inc.). One-way analysis of variance (ANOVA) was used to compare groups followed by Fisher’s least significance difference test whenever significant effects were found in ANOVA. Statistical significance was set at *p* < 0.05. Sample size is indicated in each figure legend.

## Results

**FIGURE 1:**
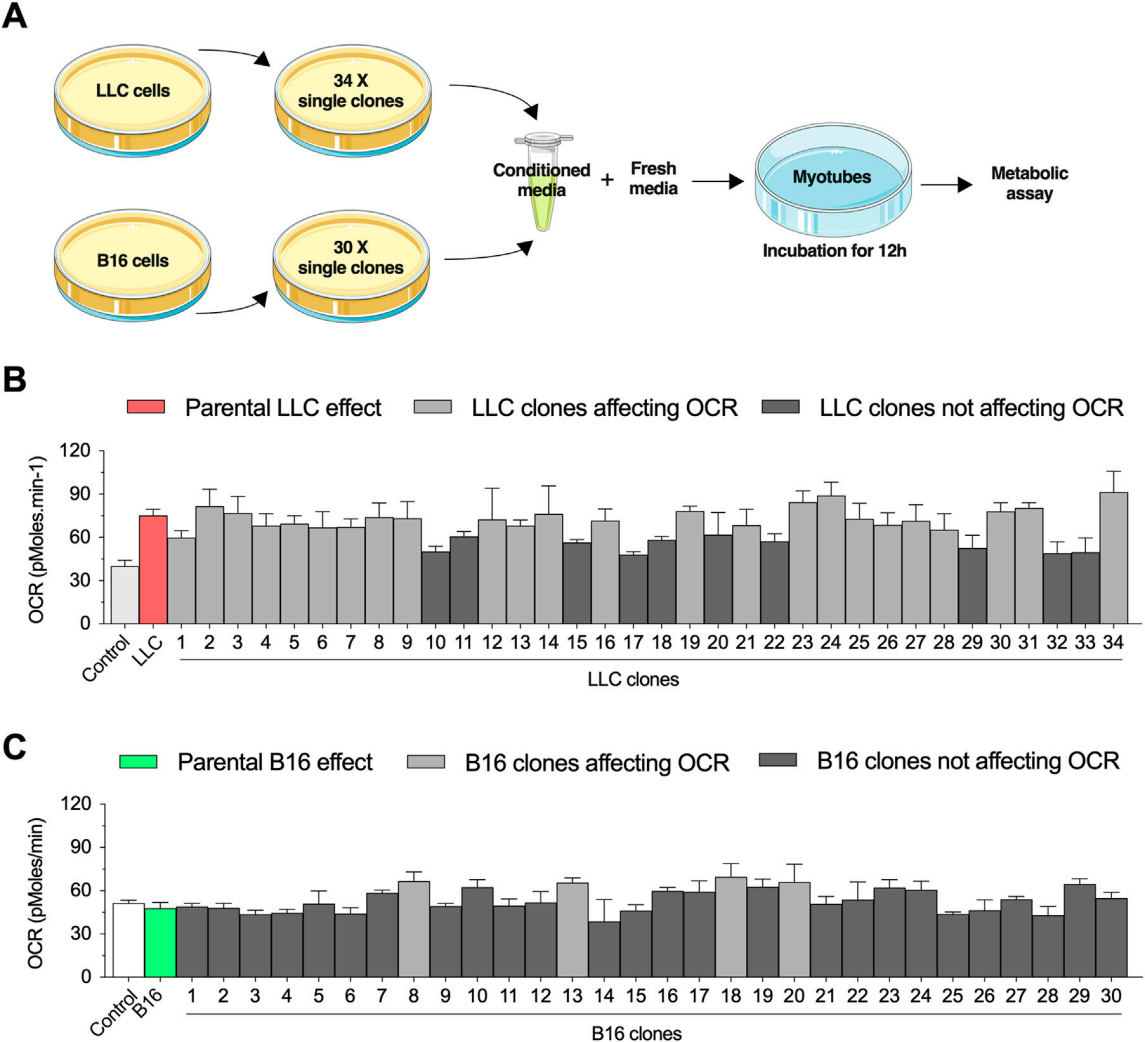
Selection of conditioned media from Lewis lung carcinoma (LLC) and B16-F10 melanoma (B16) single clones to challenge primary mouse myotubes. **(A)** Experimental design to challenge myotubes with LLC-conditioned media. All experiments are conducted by diluting the conditioned media with fresh media (ratio 1:1 with ideal final serum concentration) to avoid deprivation of critical nutrients. **(B,C)**: Basal oxygen consumption rate (OCR) in myotubes incubated for 12 h with conditioned media from different LLC **(B)** and B16 **(C)** clones. Two control conditions were applied for each cell line: 1) a healthy naïve control condition and 2) a condition treated with conditioned media from the parental cell lines. Dark gray bars indicate the clones that did not reach statistical difference when compared to naïve control condition, while light gray bars indicate the clones statistically different when compared to naïve control condition. Data are presented as mean ± standard error of the mean. *n* = 3 technical replicates for all measurements.

We have previously demonstrated that short-term exposure to LLC-conditioned media increases basal OCR in primary myotubes (Alves et al., 2020). Here, we applied a combination of cell cloning and extracellular flux experiments to study the biological variability of these effects. We first established 40 single-cell clones from the parental LLC cells and selected 34 clonal cell lines that had similar growth rates to generate LLC-conditioned media (Figure 1A). Differentiated primary mouse myotubes were incubated for 12 h with LLC-conditioned media derived from each individual clonal cell line (Figure 1A). As expected, incubation with LLC-conditioned media significantly increased basal OCR levels, although there was a notable variability in the magnitude of these effects across the different clonal cell lines (Figure 1B). Conditioned media from 10/34 clonal cell lines (black bars) did not promote statistically significant effects when compared to untreated myotubes (Figure 1B). In parallel, we performed a similar approach using the B16 cell line, which was previously shown to not affect basal OCR in primary myotubes (Alves et al., 2020). We established 30 single clonal cell lines with similar growth rates to generate B16-conditioned media (Figure 1A). While there was no effect of the parental B16-conditioned media on myotubes, 4/30 clonal lines promoted modest increases (dark gray bars) in basal OCR level (Figure 1C). Based on these findings, we selected a clonal LLC cell line (clone #10) that failed to increase myotubes OCR and a clonal B16 cell line (clone #8) that induced modest changes in myotubes OCR for further investigation.

**FIGURE 2:**
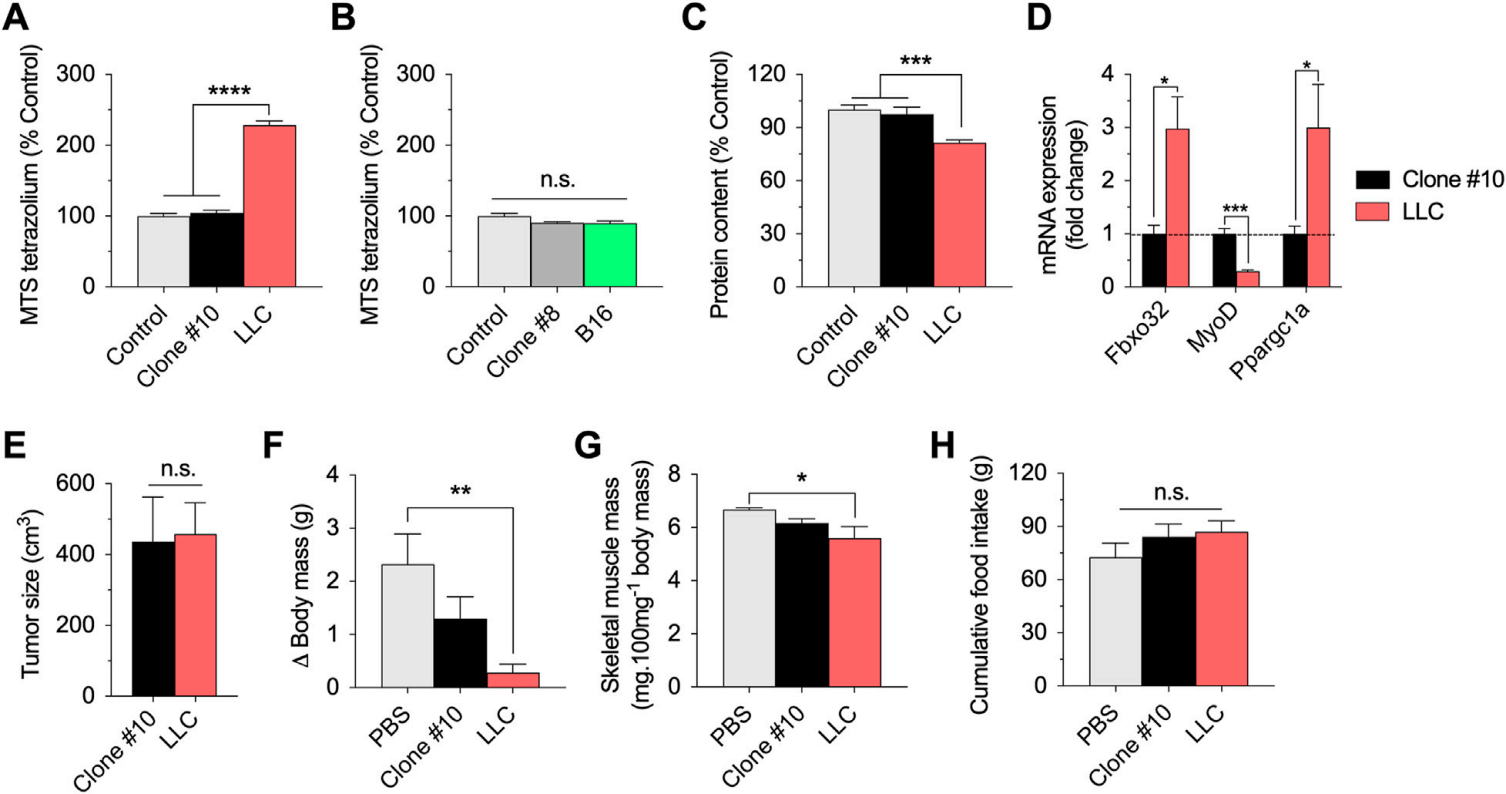
*In vitro* and *in vivo* effects of the selected LLC and B16 clones. **(A,B)**: Reduction of MTS tetrazolium compound in myotubes incubated for 12 h with parental or clonal LLC or B16-conditioned media. **(C)**: Total protein content in myotubes incubated for 48 h with parental LLC or LLC clone#10 -conditioned media. **(D)**: mRNA expression of key regulators of muscle function in myotubes incubated with parental LLC or LLC clone #10-conditioned media. Data is normalized using untreated myotubes. **(E–G)**: Tumor growth **(E)**, changes in body (carcass) mass **(F)**, sum of gastrocnemius and plantaris muscle mass normalized by initial body mass **(G)**, and cumulative food intake **(H)** in LLC tumor-bearing mice 21 days post injection. Data are presented as mean ± standard error of the mean. *n* = 3 technical replicates for all measurements. *n* = 5 mice/group (one mouse from the LLC group died before 21 days and data was not collected/plotted). *p < 0.05; **p < 0.01; ***p < 0.001; ****p < 0.001.

As an additional metabolic parameter, we evaluated the reduction of the MTS tetrazolium compound, which is produced by dehydrogenase enzymes in the presence of NADPH or NADH in metabolically active cells. Incubation with parental LLC-conditioned media remarkably increased the reduction of MTS tetrazolium. However, incubation with LLC-conditioned media derived from clone #10 showed similar values to the controls (Figure 2A). Neither parental B16-conditioned media nor B16-conditioned media derived from clone #8 affect the reduction of MTS tetrazolium (Figure 2B). Based on these results, we focused on the LLC model for the next experiments.

Long-term incubation (48 h) with parental LLC-conditioned media decreased total protein content in myotubes, suggesting cell death and/or muscle atrophy over time (Figure 2C). However, long-term incubation with LLC-conditioned media derived from clone #10 did not affect total protein content (Figure 2C). As complementary analysis for the experiments involving LLC-conditioned media, we tested the expression of key regulators of muscle function, including *Fbxo32* (*Atrogin-1*), *MyoD*, and *Ppargc1a* (*PGC1-alpha*) (Figure 2D). While incubation with parental LLC-conditioned media increased the *Atrogin-1* and *PGC1-alpha* mRNA expression, and decreased the *MyoD* mRNA expression, incubation with LLC-conditioned media derived from clone #10 had no effects in the expression of these genes (Figure 2D). Taken together, these data indicate that LLC clonal cell line #10 does not challenge myotubes like the parental LLC cell line.

Several previous studies have demonstrated that injection of LLC cells induces cancer cachexia in mice (Penna et al., 2011; Ruas et al., 2012; Kir et al., 2014; Puppa et al., 2014; Alves et al., 2019). To test the hypothesis that this established clonal cell line #10 would fail to induce similar catabolic effects *in vivo*, we compared the effects of subcutaneous injections of clonal LLC line #10 and parental LLC cells in wild-type mice. Both cell lines generated solid tumors in mice and there was no difference between groups in tumor growth at 21 days post injection (Figure 2E). At the same time point, while the tumor-bearing mice injected with parental LLC cells had significantly reduced body mass (Figure 2F) and skeletal muscle mass (Figure 2G), these effects were attenuated, although not fully prevented, in tumor-bearing mice injected with LLC cells derived from clone #10. Importantly, both groups of tumor-bearing mice had normal food intake during this time course (Figure 2H). In conclusion, these novel findings indicate that a specific clonal LLC cell line, whose conditioned media does not induce metabolic stress in myotubes, also promotes weaker body catabolism in mice.

## Discussion

Cachexia is a leading cause of death in advanced cancer patients and identifying novel therapies to counteract cancer cachexia is a major research challenge. Here, we found that early disruption of muscle oxidative metabolism is associated with cancer cachexia progression in mice. Corroborating our novel findings, previous reports showed that muscle mitochondrial dysfunction is one of the few pathways affected at multiple time points during tumor progression in mice (Blackwell et al., 2018), and LLC tumor-bearing mice have impaired mitochondrial quality control prior to muscle loss (Brown et al., 2017). We and others have previously reported that tumor-bearing rats have severe muscle atrophy exclusively in glycolytic fibers, indicating that muscle oxidative capacity determines the response to the cachectic effects (Acharyya et al., 2005; Alves et al., 2021). Moreover, aerobic exercise training improves muscle oxidative metabolism and, consequently, attenuates cancer-induced muscle wasting in a model of severe cancer cachexia (Alves et al., 2020). Considering all these pre-clinical findings, we speculate that improving muscle oxidative metabolism in cancer patients may prove to be a helpful strategy for delaying the onset of cachexia and/or mitigating the long-lasting catabolic effects induced by tumor progression. Thus, future clinical studies assessing whether muscle oxidative capacity is a significant predictor of cancer cachexia and overall survival in cancer patients are highly encouraged.

As expected, we found that LLC-conditioned media induces metabolic stress in primary myotubes and LLC injections in mice induce cancer cachexia. We expect that LLC cells produce factors such as metabolites and pro-inflammatory cytokines that directly perturb the homeostasis of myotubes as indicated by the increased basal OCR levels. Importantly, all incubations in myotubes were performed using the same final serum concentration and with addition of new media to ensure that changes in myotubes metabolism were not related to nutrient deprivation. Moreover, the OCR readout was performed after a relatively short-term exposure (12 h), a time point at which we do not observe cell death. Our single clone approach demonstrates that some LLC clonal lines lose the capacity to produce those factors that affect myotube metabolism. A similar approach was previously performed by another group to study the effects of tumor-derived factors primarily in adipose tissue and they demonstrated variability in the effects induced by different LLC clones (Kir et al., 2014). Our data also indicates variability in the effects induced by different cancer clonal cells, which is not surprising due to the heterogenous characteristic of many tumors.

In this study, a selected clonal LLC cell line that failed to induce metabolic stress in myotubes also promoted weaker catabolism in myotubes and in mice, indicating that disruption of muscle oxidative metabolism is associated with cancer cachexia progression. Additional indicators of myotubes atrophy include a 3-fold increase in *Atrogin-1* levels and a drastic decrease in *MyoD* levels. *Atrogin-1* is a E3 ubiquitin ligase induced in catabolic states (Bodine et al., 2001; Gomes et al., 2001; Cunha et al., 2012; Bodine and Baehr, 2014) and *MyoD* is a regulator of skeletal muscle differentiation downregulated in a pro-inflammatory environment such as cancer cachexia (Guttridge et al., 2000). These catabolic effects are probably induced by factors produced directly from tumors. Previous studies have demonstrated that some of these catabolic mediators could include pro-inflammatory cytokines (Argiles et al., 2018), fatty acids (Fukawa et al., 2016), microvesicles (He et al., 2014), and other specific factors such as Ataxin-10 (Schafer et al., 2016) and PTHrP (Kir et al., 2014). In this sense, our data corroborate previous studies indicating that catabolic factors secreted by LLC cells induce cancer cachexia *in vitro* and in mice.

We performed a similar approach using the B16 cell line that was previously found to not affect basal OCR in primary myotubes (Alves et al., 2020). We hypothesized that some single clone could grow and produce factors that challenge the myotube metabolism. However, only minor effects were observed with some clones on OCR and these effects were not reproducible using another metabolic readout (reduction of MTS tetrazolium). While the potential catabolic effect of B16 tumors should be further investigated, these findings are in line with our previous observations that B16-derived factors are less catabolic than LLC-derived factors, at least in the context of muscle metabolic dysfunction (Alves et al., 2020).

In summary, by conducting experiments with single clones derived from the LLC cells, we demonstrate for the first time that a specific clonal LLC cell line, whose conditioned media does not induce metabolic stress in myotubes, also fails to promote cancer cachexia in mice. These novel findings provide new insights that early disruption of muscle oxidative metabolism is associated with cancer cachexia progression and should be considered in future clinical cohort studies, trials, and practice.

## Data Availability

The raw data supporting the conclusion of this article will be made available by the authors, without undue reservation.
